# Assessing the role of interventions and climate on malaria mortality among children under five years of age: insights from two decades of data from the Health Demographic Surveillance System of Nouna, Burkina Faso

**DOI:** 10.7189/jogh.16.04080

**Published:** 2026-04-03

**Authors:** Nafissatou Traoré, Pascal Zabré, Ourohiré Millogo, Ali Sié, Penelope Vounatsou

**Affiliations:** 1 Swiss Tropical and Public Health Institute, Allschwil, Switzerland; 2 University of Basel, Basel, Switzerland; 3 Nouna Health Research Centre/National Institute of Public Health, Nouna, Burkina Faso; 4 Institut de Recherche en Sciences de la Santé/Centre National de Recherche Scientifique et Technologique, Ouagadougou, Burkina Faso

## Abstract

**Background:**

Malaria is a preventable disease that causes serious illness and death. In 2022, it remained the leading cause of death among children under five years of age in Burkina Faso, despite significant intervention efforts over the past two decades. Research on the effects of interventions and climatic factors on malaria morbidity has expanded, but their effects on malaria mortality remain unclear. We aimed to estimate the effects of interventions and lagged climatic factors on malaria mortality among children under five years of age in northwest Burkina Faso. We further evaluated the role of climatic seasonality in patterns of malaria mortality.

**Methods:**

We investigated the seasonal patterns of malaria mortality among children under five years of age and their association with climatic factors, such as rainfall and land surface temperature (LST), using wavelet analysis on mortality data from the Nouna Health Demographic Surveillance System spanning 2002–2021. Furthermore, we assessed the effects of interventions, including coverage of insecticide-treated nets (ITNs) and artemisinin-based combination therapies (ACTs), on malaria mortality alongside climate effects using Bayesian negative binomial temporal models for the period 2013–2021.

**Results:**

The lag time in the effects of climatic factors varied over time. Malaria mortality, rainfall, and LST showed a 12-month seasonal cycle throughout the years, while LST also had a six-month cycle in specific years. Rainfall lagged by 1.5 to 2 months and LST by 1 to 1.5 months, depending on the seasonal cycle and year. Rainfall was positively associated with malaria mortality (mortality rate ratio (MRR) = 1.59; 95% Bayesian credible interval (BCI) = 1.18, 1.95), LST showed a decrease in mortality (MRR = 0.68; 95% BCI = 0.52, 0.86), and ITN was associated with a reduction in mortality (MRR = 0.59; 95% BCI = 0.42, 0.79); however, ACT was not statistically important.

**Conclusions:**

We found that ITN was more effective in reducing malaria mortality than temperature, but rainfall had a greater opposing impact on increasing malaria mortality. The seasonal mortality pattern was more influenced by rainfall than by temperature. Varying climatic lag times highlight the need for adaptive strategies. Policymakers should focus on climate-informed planning, sustained ITN coverage, and reassessment of ACT strategies to further reduce malaria mortality.

Malaria is a key focus of the United Nations’ Sustainable Development Goal 3, which aims to end the malaria epidemic by 2030 [1]. This mosquito-borne disease, caused by *Plasmodium* parasites, remains a major global health issue, particularly in tropical and subtropical regions [2]. Itwas responsible for an estimated 608 000 deaths in 2022, with sub-Saharan Africa bearing the greatest burden, accounting for 233 million cases and 580 000 deaths [3]. In Burkina Faso, malaria is the leading cause of death among children under five years of age. It led to 38% of medical consultations, 63% of hospitalisations, and 18% of deaths in 2022 [4]. Since 2010, Burkina Faso’s Ministry of Health has implemented interventions, such as distributing insecticide-treated nets (ITNs), using artemisinin-based combination therapies (ACTs), indoor residual spraying (IRS), and seasonal malaria chemoprevention (SMC) for children. These efforts have significantly reduced malaria prevalence from 76.1% in 2010 to 28% in 2021 in the country [5].

Several studies have examined the effects of interventions on malaria mortality in Africa, with conflicting evidence regarding the impact of ITNs under routine conditions. Some studies report a decrease in all-cause mortality with ITN use [6,7], while others found no difference in mortality between villages with and without bed nets [8]. In Tanzania, a 10% increase in ITN coverage was linked to a 5.4% decrease in malaria deaths among children under five years of age [9]. Moreover, bed net use during pregnancy has been associated with a 33% reduction in miscarriages and stillbirths [10]. Furthermore, proper training and drug access enable mothers to effectively treat malaria episodes, reducing malaria morbidity and mortality [11]. In Burkina Faso, the mortality risk was lower in children treated with antimalarial drugs (5%) compared to those untreated (11%).

Malaria transmission, driven by its complex life cycle in mosquitoes and humans, is heavily influenced by environmental and climatic factors, which also affect the effectiveness of interventions [12]. Climatic factors like rainfall and temperature impact mosquito development and parasite growth [13,14]. Rainfall creates breeding sites for mosquitoes [15], while excessive precipitation can increase vector mortality [16]. Temperature plays a crucial role in boosting mosquito growth, biting frequency, and accelerating parasite development [17]. High temperatures also influence water availability, potentially leading to drier conditions [18-20]. Studies from Malaria Transmission Intensity and Mortality Burden sites in Tanzania, Kenya, and Ghana have found a strong association between child mortality, control interventions, rainfall, and temperature [7,21,22].

The Nouna Health and Demographic Surveillance System (HDSS) is a well-established research platform in Burkina Faso, which routinely collects longitudinal data on cause-specific mortality and household-related indicators, including malaria interventions [23]. In Nouna, malaria-related child mortality spikes during the rainy season [24], and although few studies have explored spatial variations in malaria mortality in this setting [25], Nicholas *et al.* found significant lag effects of temperature and rainfall [26]. However, they did not account for malaria vector control measures, such as bed nets and ACT, which could significantly impact mortality and interact with climatic factors. Other studies have shown a strong link between interventions and climatic predictors [27]. The effects of interventions in reducing malaria incidence in Burkina Faso are well recognised, but more research is needed to assess their effects on malaria-related deaths.

We aimed to estimate the effects of control interventions and lagged climatic factors on malaria-related deaths in children under the age of five in Nouna. We also explored the seasonality of malaria mortality and assessed how fluctuations in the seasonality of climatic factors over time affected mortality dynamics.

## METHODS

The analysis utilised high-quality monthly malaria mortality data from the Nouna HDSS, collected from 2002 to 2021. We employed various formulations of Bayesian negative binomial temporal models to quantify the effects, and conducted wavelet analysis to better understand the impact of climate variability on malaria mortality.

### Study area

The Nouna Health Research Centre (CRSN), one of the four research institutes under the Burkina Faso Ministry of Health, has operated the HDSS since 1992 in the Nouna health district catchment area in northwest Burkina Faso, 300 km from the capital city Ouagadougou. The HDSS area covers approximately 1775 km^2^, has a sub-Saharan climate with an average annual rainfall of 800 mm, occurring mainly from May to September, and fairly stable daily minimum temperature (20–28.1°C) and maximum temperatures (29.5–37.2°C) throughout the year. The region includes plateaux with gentle slopes and several small semi-permanent streams. The HDSS serves approximately 90 000 people in 11 750 households across 58 villages and the town of Nouna [28].

### Data sources

#### Malaria death data and control interventions data

We used malaria mortality data from 2002 to 2021, obtained from the longitudinal cause-of-death data of the Nouna HDSS. Trained non-medical fieldworkers determined causes of death using verbal autopsy (VA), collecting information from caregivers about the deceased’s characteristics and symptoms through structured questions [29]. The VA is typically conducted at least three months after the death to allow for mourning, using identification information from the HDSS [30].

We aggregated mortality data at the sub-district level to link it with malaria control intervention data. We extracted monthly proportions of children using ITNs and those treated with ACT for malaria from the District Health Information System (DHIS2) [30]at the sub-district level for 2013 to 2021. The DHIS2 has managed national health data since 2013. The Nouna HDSS covers 58 villages across five sub-districts: Bourasso, Nouna, Dokui, Sono, and Barakuy.

#### Climatic data

We extracted monthly average land surface temperatures from the Fifth Generation of the European Centre for Medium-Range Weather Forecasts Atmospheric Reanalysis of the Global Climate [31] at a spatial resolution of 11 × 11 km^2^ and a temporal resolution of eight days. We obtained monthly average rainfall data from the Climate Hazards Group Infrared Precipitation with Station [32]at 5.6 × 5.6 km^2^ spatial resolution and a five-day temporal resolution for 2002 to 2021. We extracted climatic factors at the sub-district level to align with the monthly mortality data.

### Statistical analysis

We divided the number of malaria deaths among children under five years of age in a given village and month by the total person-time of observation (expressed as person-years of observation or PYO) to calculate the village- and month-specific under-five malaria mortality rates. We defined person-time as the time spent in the HDSS from enrolment to exit through death, out-migration, loss to follow-up, or the end of the observation period (December 2021). We presented mortality rates per 1000 PYO.

We employed a multi-step approach combining wavelet analysis and Bayesian temporal modelling to evaluate the impact of climate variability and interventions on malaria transmission dynamics.

We used wavelet analysis to detect and characterise the temporal and frequency relationships between malaria mortality and climatic factors from 2002 to 2021. This method is particularly suitable for dealing with non-stationary time series data, where relationships may change over time [33]. We used the wavelet power spectrum to visualise the magnitude of variation in malaria mortality, rainfall, and LST across different time scales and frequencies, thereby identifying periodic patterns and their changes over time. We calculated wavelet cross-coherence to quantify the correlation between two non-stationary time series, such as malaria and rainfall or LST. It provides information about the specific frequencies at which the two time series are correlated, as well as the time intervals during which this correlation occurs. We estimated the phase difference to assess delays in the relationship between the malaria mortality and climatic factors time series, evaluating how much time one series leads or lags behind the other. It is expressed in radians, from −π to +π, and displayed as arrow angles. An angle of 0 radians (an arrow pointing right) means the malaria time series and that of a climatic factor are in perfect synchrony (peaking and dipping simultaneously). An angle of π radians (an arrow pointing left) means the two time series are perfectly out of synchrony (when one peaks, the other dips). Angles between 0 and π (or 0 and -π) indicate varying degrees of lead or lag between the two series. We used a 5% significance level to assess the statistical significance of the observed patterns and carried out the wavelet analysis using the wavelet Comp package in *R*, version 4.3.1. (R Core Team, Vienna, Austria).

While wavelet analysis identifies patterns and lags, it does not quantify the strength or statistical significance of these relationships in a predictive framework. We therefore conducted Bayesian temporal models to simultaneously assess the effects of climatic factors and interventions on malaria mortality from 2013 to 2021. We created seven monthly lag variables for each climate factor by averaging values over the following periods: the current month in which malaria mortality was reported (lag 0), one month prior (lag 1), two months prior (lag 2), three months prior (lag 3), four months prior (lag 4), the current and previous month (lag 01), the current and two previous months (lag 012), and the current and three previous months (lag 0123). The choice of lag time for rainfall and temperature aligns with both the biological timelines of malaria transmission and Burkina Faso’s climatic patterns [34,35]. These lags capture the mosquito life cycle, parasite development, and disease progression. We selected a maximum lag of 4 months to correspond to the season period in Burkina Faso, typically lasting from June to September [36]. This allowed us to capture the full extent of seasonal malaria transmission, as peak incidence occurs during and immediately after the rainy season. To avoid collinearity, we calculated the variance inflation factor (VIF) between the lagged variables, and removed those with a VIF higher than five.

We employed three formulations of Bayesian negative binomial (NB) models: NB with an exchangeable random effect; NB with a random effect following an autoregressive moving average (ARMA (p, q)) process; and NB generalised autoregressive moving average (GARMA (p, q)) model. We fitted several models with different combinations of p and q orders, and we selected the model with the smallest deviance information criterion (DIC). We also used DIC to identify the best lag combination for rainfall and temperature associated with malaria mortality in children under five. We modelled all possible combinations of the remaining lag variables from the collinearity test. We used the total pyo of observation as an offset and standardised predictors by subtracting the mean and dividing by their standard deviation to compare the effects.

We implemented models in Just Another Gibbs Sampler, and estimated parameters using the Markov Chain Monte Carlo algorithm [37] with 200 000 iterations. To determine convergence of the algorithm, we utilised the Gelman-Rubin diagnostic [38]. We summarised the parameter estimates by computing the posterior medians of the mortality rate ratio (MRR) and their corresponding 95% Bayesian Credible Intervals (BCIs). We considered a predictor’s effect statistically important if its 95% BCI did not include one (**Online Supplementary Document**).

## RESULTS

### Descriptive analysis

We found a decrease in overall malaria mortality among children under the age of five (Figure S1 in the **Online Supplementary Document**). We observed a slight increase in LST and a reduction in rainfall from 2002 to 2021, as well as a significant dip in rainfall in 2011. At the same time, we noted a decrease in mortality and an increase in LST. The average annual mortality rate was 1.72 per 1000 PYO over the study period. The highest mortality rate was 7.60 per 1000 PYO in 2002, and the lowest was 0.95 per 1000 PYO in 2021 (Table S1 in the **Online Supplementary Document**). Additionally, we observed an average of 123 mm of rainfall per year, while the annual average temperature was 29.13°C during 2002–2021.

Rainfall levels increased between 2013 and 201, decreased in 2016, and then increased again in 2017 and remained at that level until 2021 (Figure S2 in the **Online Supplementary Document**). The average ITN use coverage was 0.36 per year from 2013 to 2021, while ACT coverage was 0.93 (Table S1 in the **Online Supplementary Document**). From 2013 to 2017, ITN use remained consistent, with an upward trend observed from 2018 onwards. ACT coverage stayed relatively stable, though a dip occurred between 2015 and 2016 (Figure S2 in the **Online Supplementary Document**)

**Figure 1:**
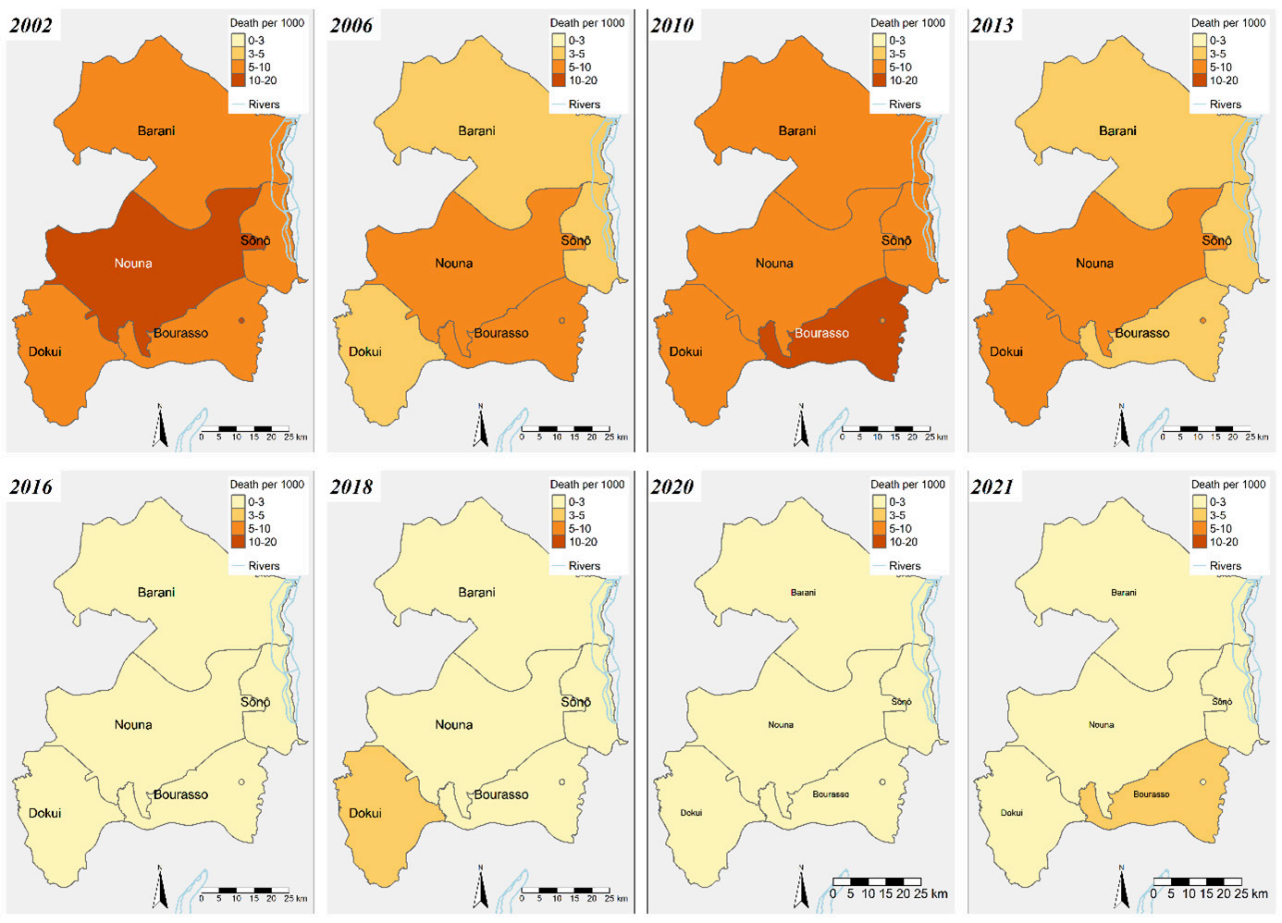
Geographical distribution of mortality rates per 1000 PYO in children under five years of age across the five sub-districts within the HDSS from 2002 to 2021.

We also presented the geographical distribution of mortality rates in children under five over 20 years (**Figure 1**). Overall, we saw a slight variation in mortality across the sub-districts within the HDSS. Mortality rates declined from 2002 to 2013, with a notable spike in 2010 across all sites. After 2013, we observed a significant decrease in mortality. The highest mortality rates occurred from July to November, with the lowest rates observed in March and April, while August and November had the highest number of deaths across the HDSS (Figure S3 in the **Online Supplementary Document**).

### Temporal dynamics of malaria mortality in association with climatic factors from 2002 to 2021

**Figure 2:**
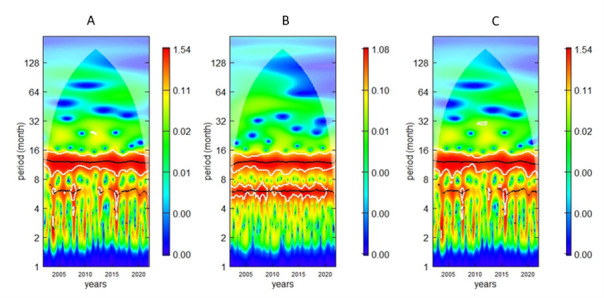
Wavelet spectrum power level of mortality per 1000 PYO in children under five years of age from 2002 to 2021. **Panel A.** Malaria mortality. **Panel B.** Land surface temperature. Panel C. Rainfall. The cone of influence (COI), where edge effects might influence the analysis, is depicted as a lighter shade. Patterns below the cross-hatched region are considered statistically significant. The colour code ranges from blue (low values) to red (high values), indicating increasing significance levels. The white lines outline areas of significance.

We observed a 12-month cycle of malaria mortality from 2002 to 2021 (**Figure 2**, Panel A). LST shows two periodicities: a 12-month cycle of rainfall from 2002 to 2021 and a 6-month cycle during 2002–2009 and 2011–2020, and a 12-month cycle from 2002–2021 and a 6-month seasonality in 2009 and 2016 (**Figure 2**, Panels B and C). Moreover, we estimated the average wavelet power at the 5% level of significance for mortality, LST, and rainfall. The average power plot highlighted a significant and strong 12-month cycle of mortality. We found the 12-month and 6-month temperatures significant at the 5% level. We found that the 12-month seasonality of rainfall was significant across all time intervals. However, we found the average rainfall power at a 6-month periodicity insignificant and dominated by the 12-month cycle (Figure S3 in the **Online Supplementary Document**, Panels A–C).

**Figure 3:**
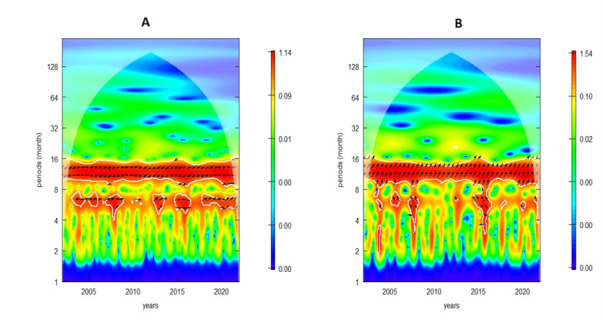
Cross-coherence of malaria mortality. **Panel A.** Land surface temperature. **Panel B.** Rainfall. The cone of influence (COI), where edge effects might influence the analysis, is depicted as a lighter shade. Patterns below the cross-hatched region are considered statistically significant. The colour code ranges from blue (low values) to red (high values), indicating increasing significance levels. The white lines outline areas of significance.

We observed a significant and negative correlation between malaria mortality and LST at a 12-month periodicity (**Figure 3**). Rainfall was in phase with malaria mortality, as indicated by the right-facing arrows, highlighting a positive association. Moreover, both LST (**Figure 3**, Panel A) and rainfall (**Figure 3**, Panel B) precede malaria mortality. We also observed varying delay times; rainfall precedes mortality by 1.5 to 2 months for a 12-month periodicity (**Table 1**). Similarly, temperature precedes mortality by 1 to 1.5 months at both the 6-month and 12-month cycles.

**Table 1:** Summary of lag times in the effect of climatic factors on malaria mortality and seasonal scales over 2002–2021

	Month and year intervals
**Seasonal scales**	
Rainfall	12 (2002–2021)
LST	12 (2002–2021); 6 (2002–2009, 2011–2020)
Malaria mortality	12 (2002–2021)
		**Lag times**	
Rainfall over mortality	
*12-month period*	1.5 (200–2009); 2 (2010–2021)
LST over mortality	
*6-month period*	1.5 (2002–2015, 2020); 1 (2016–2019, 2021)
*12-month period*	1.5 (2002–2009); 2 (2010–2021)

### Effect of interventions and climatic factors on malaria mortality from 2013 to 2021

There were 20 combinations of rainfall and temperature lags for each of the three models (exchangeable, ARMA, and GARMA) (**Table 2**). We found the combination of rainfall in the two previous months (lag 2) and LST in the previous month (lag 1) optimal for all models. The GARMA (1, 1) model had the lowest DIC, making it the best model with the best combination of lags.

**Table 2:** Deviance information Criterion values obtained from different formulations of Bayesian negative binomial models (exchangeable random effect, ARMA (1, 1) random effect and GARMA (1, 1) model) with different combinations of lag times in the LST and rainfall

Rainfall lags	LST lags	Exchangeable	ARMA (1, 1)	GARMA (1, 1)
rain_0	LST_0	2320.72	2319.73	2313.71
rain_0	LST_1	2319.56	2320.65	2312.55
rain_0	LST_2	2325.10	2324.40	2321.60
rain_0	LST_3	2326.59	2326.59	2326.59
rain_0	LST_4	2324.38	2324.38	2324.38
rain_1	LST_0	2321.30	2317.35	2308.35
rain_1	LST_1	2323.90	2319.94	2311.94
rain_1	LST_2	2324.78	2321.45	2315.74
rain_1	LST_3	2320.61	2320.61	2320.61
rain_1	LST_4	2318.95	2323.95	2319.95
rain_2	LST_0	2324.83	2316.83	2309.83
rain_2	LST_1	2316.71	2311.33	2302.59*
rain_2	LST_2	2325.40	2321.72	2315.50
rain_2	LST_3	2319.43	2319.43	2319.43
rain_2	LST_4	2318.94	2319.95	2317.55
rain_3	LST_0	2321.98	2318.95	2314.95
rain_3	LST_1	2325.21	2316.21	2316.21
rain_3	LST_2	2326.30	2319.60	2324.20
rain_3	LST_3	2326.95	2330.92	2328.95
rain_3	LST_4	2325.56	2326.32	2325.56
rain_4	LST_0	2328.92	2330.89	2314.92
rain_4	LST_1	2327.30	2326.50	2316.30
rain_4	LST_2	2329.00	2333.20	2331.10
rain_4	LST_3	2335.84	2333.78	2330.81
rain_4	LST_4	2339.28	2331.32	2325.23

ARMA – autoregressive moving average, GARMA – generalised autoregressive moving average

The results of the final GARMA (1, 1) model simultaneously assess the effects of interventions and climatic factors on malaria mortality in the Nouna HDSS (**Table 3**), which indicate that rainfall in the two previous months, LST in the previous month, and bednet use were statistically important and associated with malaria mortality in children under five. An increase in rainfall by 1mm was associated with a 59% increase in mortality rate (MRR = 1.59; 95% BCI = 1.18-1.95). An increase of 1°C in LST was related to a 32% decrease in mortality rate (MRR = 0.68; 95% BCI = 0.52-0.86). Increased bednet use was also associated with a 41% decrease in malaria deaths (MRR = 0.59; 95% BCI = 0.42-0.79), while the association withACT coverage was not statistically significant. The model’s dispersion parameter was low (0.16), indicating that the data exhibit more variability than would be expected under the Poisson distribution.

**Table 3:** Posterior median and 95% Bayesian credible Intervals (BCIs) of the GARMA (1, 1) negative binomial model

	Posterior median (95% BCI)
**Predictors**	**MRR**
Rain_2	1.59 (1.18, 1.95)*
LST_1	0.68 (0.52, 0.86)*
Bednets	0.59 (0.42, 0.79)*
ACTs coverage	1.15 (0.84, 1.49)
**Temporal parameters**	**Estimates**
Autocorrelation coefficient	0.62 (0.46, 0.78)
Moving average coefficient	−0.12 (−0.32, 0.03)
Dispersion	0.16 (0.13, 0.19)

ACT – artemisinin-based combination therapies, BCI – Bayesian credible intervals, LST – land surface temperature, MRR – mortality rate ratio

*Statistically significant.

## DISCUSSION

We employed Bayesian negative binomial temporal models to assess the effect of malaria interventions and climatic factors on malaria mortality in children under five years of age within the Nouna HDSS. We analysed the temporal dynamics of mortality associated with climatic factors using wavelet analysis.

Drawing on surveillance data from 2002 to 2021, we found a considerable decrease in mortality among children under five years of age. During this period, the temperature increased, and rainfall slightly decreased. From 2013 to 2021, we found that ACT coverage remained almost the same, while bed net use coverage slightly increased.

The wavelet analysis revealed a negative association between temperature and malaria mortality, while we observed a positive association between rainfall and malaria mortality. Furthermore, we found similar results in studies conducted in Sri Lanka and South Africa [31,39], demonstrating that an increase in temperature leads to a decrease in malaria occurrence. The wavelet analysis identified a significant 12-month cycle of malaria mortality from 2002 to 2021. Temperature exhibited two noticeable periodicities of 6 months and 12 months throughout all the years, while rainfall showed only a significant 12-month cycle over the same period, which is consistent with the results of other studies [39,40]. Additionally, the periodic pattern of malaria mortality is more closely related to rainfall than to temperature, suggesting that rainfall has a greater impact on malaria transmission. Similar findings have been reported in South Africa [41,42], as well as in East Africa [43–45] and Ghana [46].

The cross-coherence analysis highlighted a significant correlation at a 12-month cycle between both climatic factors and mortality. Rainfall led to malaria mortality by 1.5 to 2 months, while temperature led to it by 1 to 1.5 months, depending on the seasonal cycle and year. The Bayesian temporal models estimated a 2-month lag for rainfall and a 1-month lag for LST. Due to the stationarity assumption, these models are unable to estimate varying lag times over the years. Similar results related to the estimation of lag times have been found in other studies [47–49] that used stationary statistical models. For instance, a study in the KEMRI HDSS demonstrated that an increase in temperature was associated with an increase in malaria mortality, with a lag of 13-16 weeks in Ghana [21], and rainfall has been shown to significantly affect malaria mortality after a lag of nine weeks [22]. This period coincides with the theoretical vector-parasite-host cycle under optimal conditions, assuming the first blood meal of *Anopheles* is from an infected human, and the temperature is at least 25°C [21,22,50]. Another study in West Africa also found higher malaria mortality rates during the rainy season compared to the dry season [29]. In Mozambique, a strong correlation between malaria mortality and rainfall 6–8 weeks prior has been reported [51]. Nyawanda *et al.* also identified a two-month lag between rainfall and mortality in Kenya [52]. In South India [53] and Northern Uganda [54], rainfall was found to lag behind malaria cases by 1–2 months. Additionally, an increase in monthly malaria mortality was positively associated with monthly precipitation at a one-month lag in China [55]. However, a study in central India found no association between rainfall and malaria mortality [56].

The results from the Bayesian GARMA model revealed a significant effect of LST, rainfall, and bednet use on malaria mortality. It was also found that bednet coverage was associated with a reduction in mortality among children under five years old in the Nouna HDSS. Similar findings have been observed in previous studies in Africa [7,57]. For instance, in the Ifakara HDSS, bednet coverage was associated with a 12% reduction in malaria mortality among children under five years of age [9]. In research conducted in Africa and Papua New Guinea, bednets have been found to reduce malaria transmission [58,59], severe malaria [60], and malaria mortality in children under five [61]. Bednets physically prevent mosquitoes from coming into contact with humans while they sleep. The netting material serves as a barrier, making it difficult for mosquitoes to reach and bite people inside the net [62]. Additionally, the pyrethroids in the bednets repel mosquitoes, causing them to avoid the area around the net [62]. Therefore, when bednets are used at high levels of coverage within a community, they can reduce the density and lifespan of mosquito populations, offering protection not only to those sleeping under the nets but also to others who may not be using bednets [62].

We found that ACT coverage was not related to malaria mortality among children under five years of age. Several explanations could account for this. For instance, incomplete adherence to treatment prescription can lead to treatment failure and an increased risk of severe infection, including death. The effectiveness of ACT coverage might also be compromised if parasite resistance exists, rendering the treatment less effective in preventing severe cases and deaths. It has been reported that malaria parasites such as *P. falciparum, P. vivax,* and *P.malariae* have developed resistance to antimalarial drugs globally [63]. Recent studies in Cambodia [64] and Southeast Asia [65] have shown resistance to artemisinin. Additionally, one study reported that increased susceptibility to new malaria infections might impact the overall efficacy of ACT. The extent of ACT coverage and adherence could be reduced among partially immune individuals, as many infections might be asymptomatic, leading to a higher likelihood of incomplete treatment [66].

The limitations of this study include the possibility of under-reporting death cases and the lack of malaria intervention data linked to these deaths in the Nouna HDSS. Furthermore, this research excluded socioeconomic variables, such as distance to healthcare facilities, household income, access to healthcare services, and education levels, which could significantly influence malaria mortality, due to concerns about the quality and completeness of the data available within the HDSS. Inconsistent and incomplete data could lead to unreliable results [67], potentially obscuring the true relationship between these factors and malaria mortality. Moreover, conducting VAs three months after death may introduce recall bias, as respondents may not accurately recall symptoms or events prior to death. Additionally, missing VAs due to non-participation may lead to selection bias. Specifically, deaths without VA data may differ systematically from those included, potentially underestimating malaria-related mortality in endemic regions and overestimating it in areas with lower prevalence. Despite these limitations, this study is the first to assess the combined effect of interventions and climatic factors on malaria-specific mortality in children under five in the Nouna HDSS.

## CONCLUSIONS

We described the relationship between malaria interventions, lagged climatic factors, and malaria mortality among children under five in the Nouna HDSS. We found interventions, particularly ITN use, to be strongly associated with reduced mortality, while ACT coverage showed no significant impact. Higher temperatures were associated with lower malaria mortality, while increased rainfall was linked to higher mortality, highlighting the influence of climatic conditions on disease transmission. Rainfall showed a significant 12-month cycle correlation with malaria mortality, and temperature demonstrated consistent associations at 6-month and 12-month cycles, indicating that temperature impacts vary over time. Rainfall lagged by 1.5 to 2 months and LST by 1 to 1.5 months, depending on the seasonal cycle and year.

These findings underscore the critical need for climate-informed malaria control strategies. Policy makers should prioritise targeted ITN distribution aligned with rainfall forecasts, initiate campaigns 1–2 months before the onset of the rainy season, and strengthen community-level interventions to improve ITN use. Given the non-significant impact of ACT coverage, there is a need of reassessement of its strategies by conducting regular efficacy monitoring, especially in high transmission areas and during peak seasons, strengthening supply chain management to ensure consistent availability, and implementing rotation strategies for different ACT combinations to mitigate the risk of drug resistance. In addition, developing robust early warning systems that integrate climate data can further enhance malaria control efforts. By adopting this comprehensive, climate-responsive approach and optimising both ITN and ACT interventions, stakeholders can further reduce malaria mortality and improve child health outcomes in vulnerable regions.

## Additional material


Online Supplementary Document
