# Supplementary material for: Assessing the role of interventions and climate on malaria mortality among children under five years of age: insights from two decades of data from the Health Demographic Surveillance System of Nouna, Burkina Faso
Source: J Glob Health. 2026 Apr 3;16:04080. doi: 10.7189/jogh.16.04080 (PMC13045928; doi:10.7189/jogh.16.04080)
Supplement: Online Supplementary Document [file jogh-16-04080-s001.pdf]

Supplement to: Traoré N, Zabré P, Millogo O, Sié A, Vounatsou P. Assessing the role of interventions and climate on malaria mortality among children under five years of age: insights from two decades of data from the Health Demographic Surveillance System of Nouna, Burkina Faso. J Glob Health. 2026;16:04080.

1-Tables and figures

Table S1: Climatic data sources

Source	Climatic data	Period	Spatial resolution	Temporal resolution
CHIRPS	Rainfall	2021	5.6 × 5.6 km^2	5 days
MODIS	Day-time land surface temperature	2021	1 x 1 km^2	8 days
MODIS	Night-time land surface temperature	2021	1 x 1 km^2	8 days
MODIS	Night light	2021	5 x 5 km^2	1 year
MODIS	Crop coverage	2021	5 x 5 km^2	1 year
MODIS	Forest coverage	2021	5 x 5 km^2	
MODIS	Shrubs coverage	2021	5 x 5 km^2	
MODIS	Distance to permanent water body	2021	1 x 1 km^2	1 year
SRTM	Altitude	2021	NA	NA
WorldPop	Population data	2021	1 x 1 km^2	NA

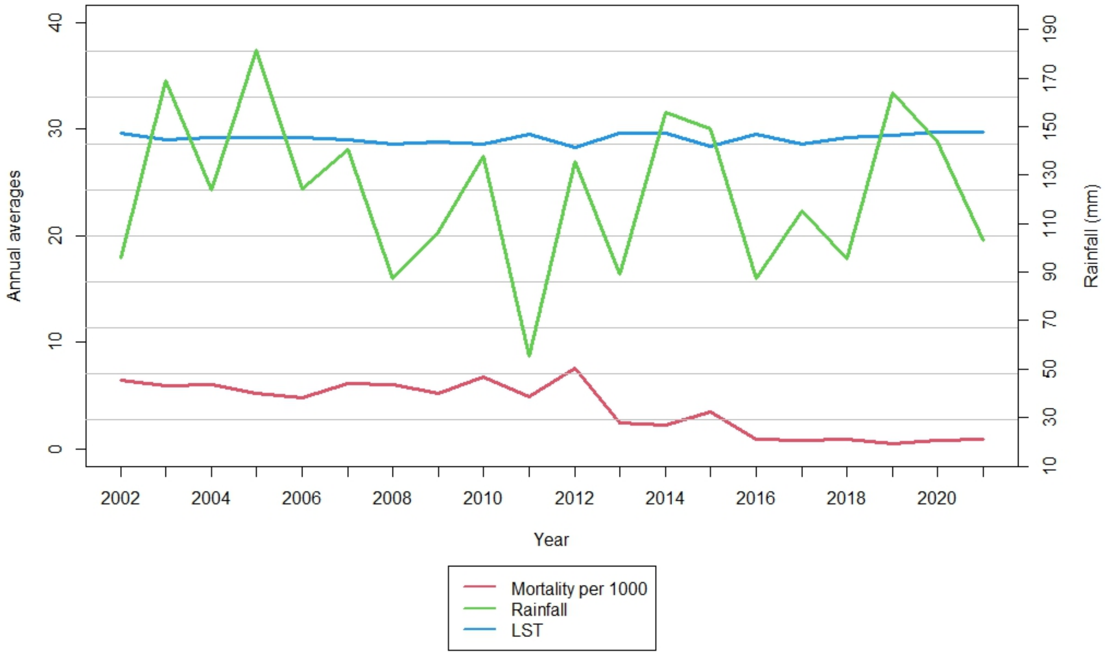

Figure S1: Trends in annual averages of monthly malaria mortality rates (per 1000 PYO) in children under five, rainfall and land surface temperature from 2002 to 2021 in the Nouna HDSS.

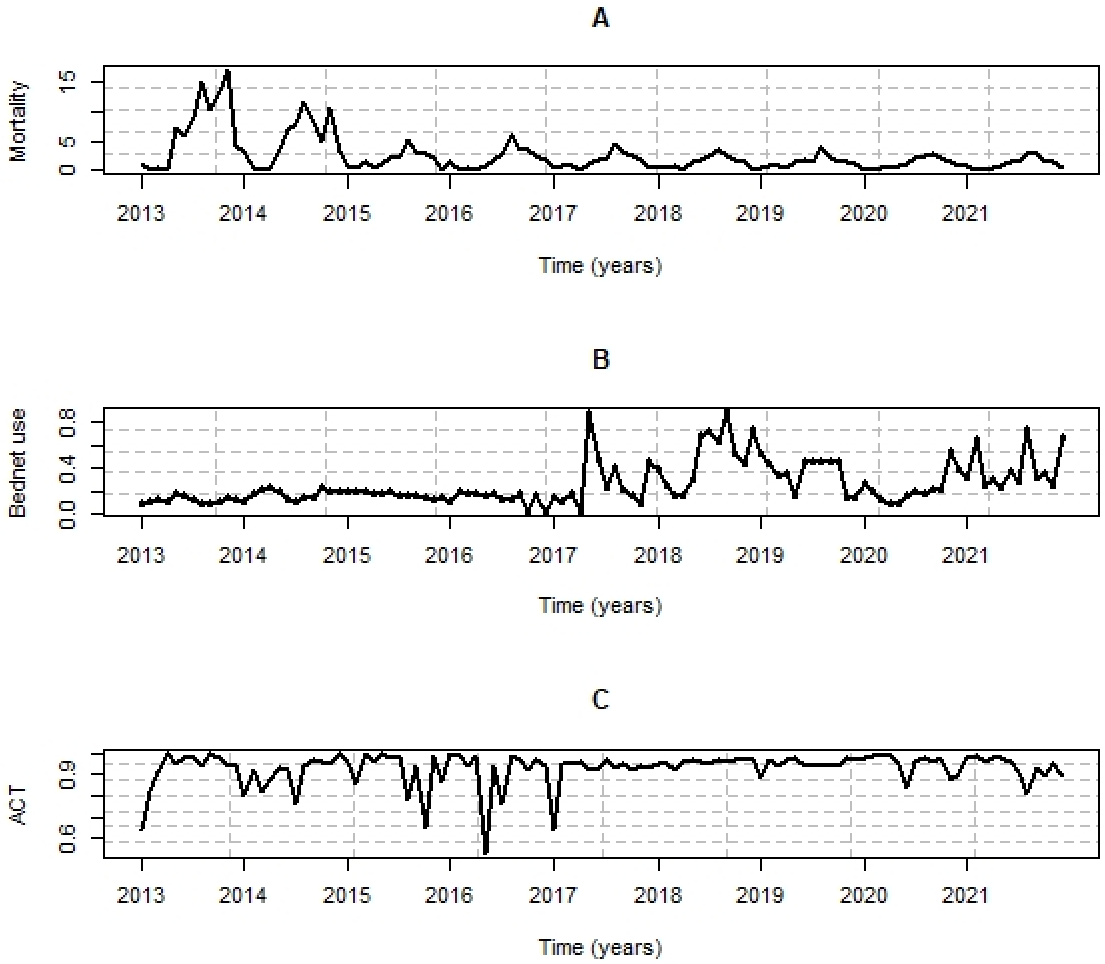

Figure S2. Time series of mortality rates per 1000 PYO during 2013–2021. **Panel A.** Malaria mortality. **Panel B.** ITN coverage. **Panel C.** ACT coverage.

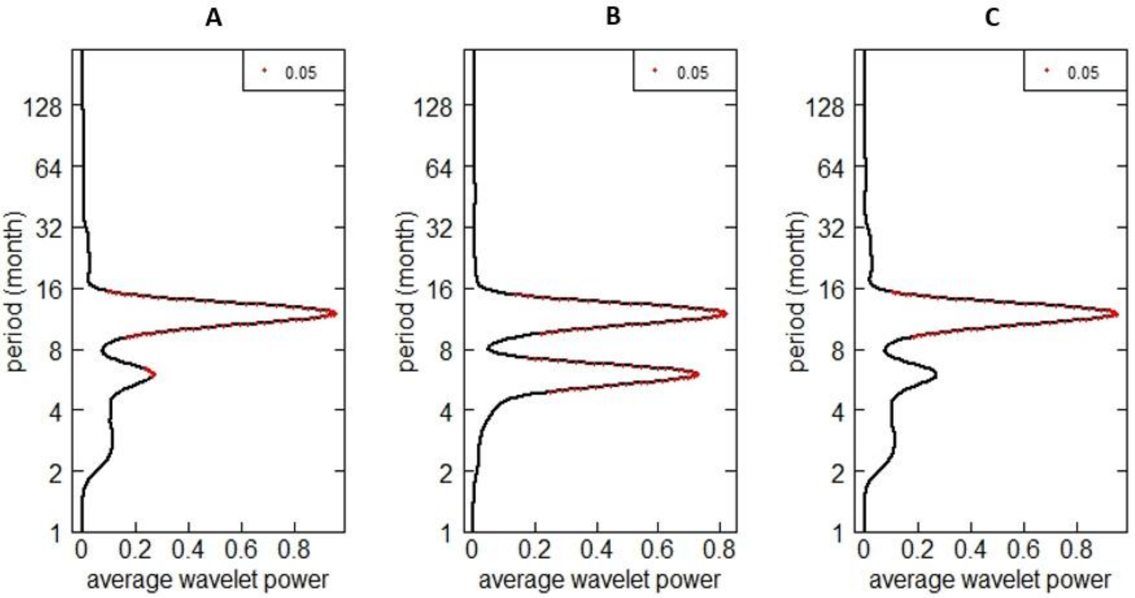

Figure S3. Average wavelet power of mortality per 1000 PYO in children under five years of age. **Panel A.** Malaria mortality. **Panel B.** Land surface temperature. **Panel C.** Rainfall.

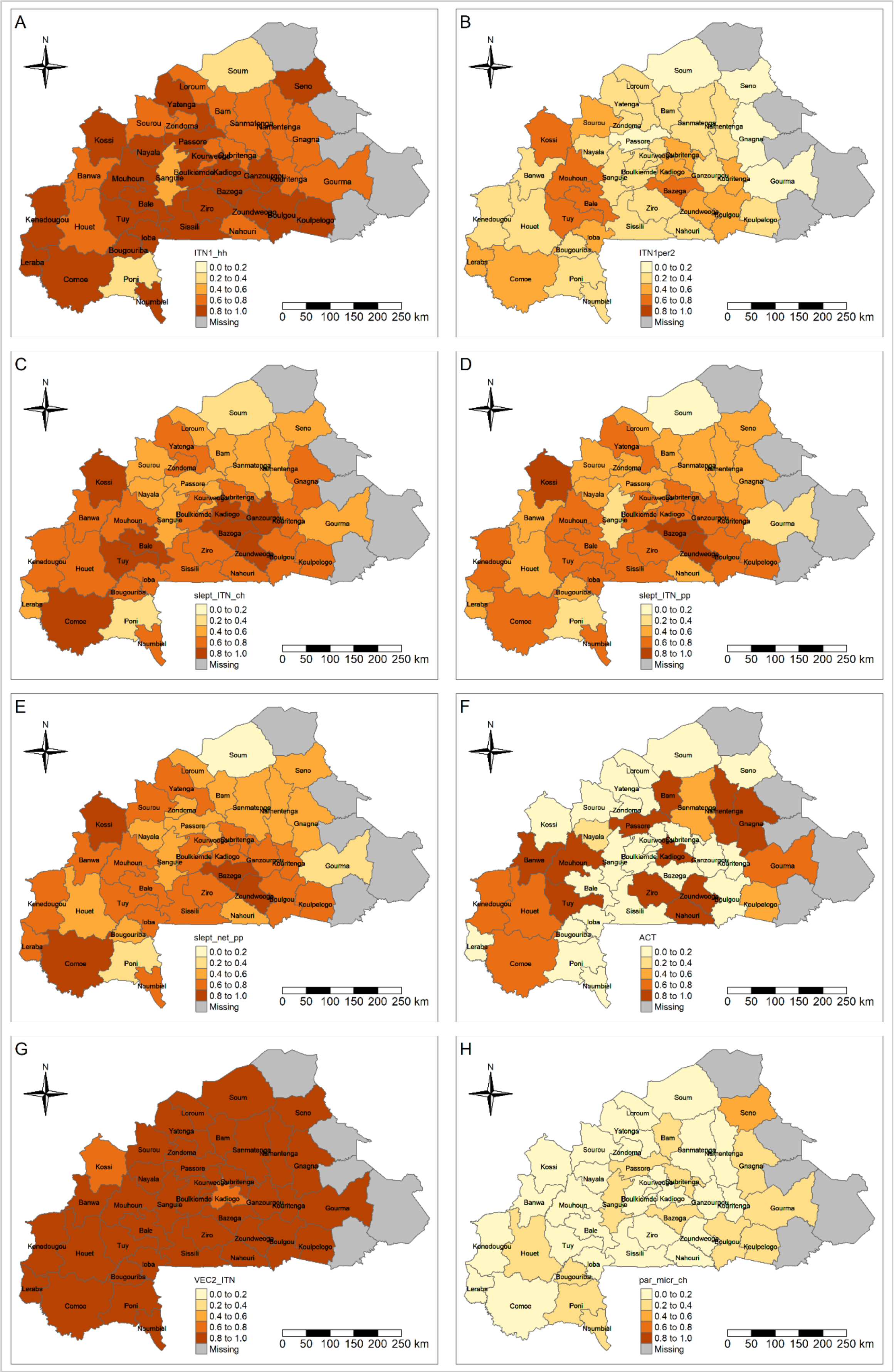

Figure S4: Interventions coverage by provinces

2- Models formulations

A.Bayesian variable selection

To choose the most important predictor and functional form that explains the maximum variation in parasitaemia odds change, Bayesian variable selection using stochastic search was implemented for both adjusted and unadjusted models. For the each covariate X_p, a categorical indicator parameter I_p was introduced to represent exclusion of the variable from the model $(I_p = 0)$, inclusion in linear $(I_p = 1)$ or categorical $(I_p = 2)$ forms. I_p has a probability mass function $\prod_{j=0}^{2} \pi_j^{\delta_j(I_p)}$, where π_j denotes the inclusion probabilities of functional form j (j=0,1,2) so that $\sum_{j=0}^{2} \pi_j = 1$ and $\delta_j(.)$ is the Dirac function, $\delta_j(I_p) = \begin{cases} 1, & \text{if } I_p = j \\ 0, & \text{if } I_p \neq j \end{cases}$. A spike and slab prior distribution was assumed for the regression coefficients. In particular for the coefficient β_p of the corresponding variable X_p in linear form, we assumed $\beta_p \sim \delta_1(I_p)N(0, \tau_p^2) + \left(1 - \delta_1(I_p)\right) N(0, \vartheta_0 \tau_p^2)$ that is a non-informative prior for β_p if X_p is included in the model in linear form (slab) and an informative normal prior shrinking β_p to zero (spike) if X_p is excluded from the model, setting ϑ_0 to be a large number, e.g, 10^5. Likewise, for the coefficients $\{\beta_{p,l}\}_{l=1,..,L}$ corresponding to the categorical form of X_p with L categories, $\beta_{p,l} \sim \delta_2(I_p)N(0, \tau_{p,l}^2) + (1 - \delta_2)N(0, \vartheta_0 \tau_{p,l}^2)$ was assumed. For inclusion probabilities, a non-informative Dirichlet distribution was adopted with hyper parameter $\alpha = (1,1,1)^T$, that is, $\boldsymbol{\pi} = (\pi_0, \pi_1, \pi_2)^T \sim \text{Dirichlet}(3, \alpha)$. We also assumed inverse Gamma priors for the precision hyper parameters τ_p^2 and $\tau_{p,l}^2$, $l = 1, \dots, L$.

B. Geostatistical model with spatially varying interventions effects

In order to estimate the intervention effects at the subnational level and account for potential interactions with endemicity levels, a geostatistical model with spatially varying regression coefficients was fitted in which we estimated malaria intervention effects at provincial level. We consider only the important interventions from the variable selections. The model was expressed as:

$\mathrm{logit}(\pi_{i'}) = \boldsymbol{\beta}^T X_{i'} + \alpha_1(\mathrm{A}_{i'})\mathrm{ITNownership}_{i'} + \alpha_2(\mathrm{A}_{i'})\mathrm{ITNuse}_{i'} + u_{i'} + v_{i'}$, where $X_{i'}$ is the set of climatic predictors. The effects of interventions are defined at provincial level and denoted as $\alpha_\mathrm{k}(\mathrm{A}_{i'}), (k = 1,2,3)$ where $\mathrm{A}_{i'}$ is the province where i' falls. Each $\alpha_k(\mathrm{A}_{i'})$ was considered to be the sum of a conditional autoregressive effect that takes into account the similarity of the effects across the provinces and an independent random component. We assumed that the first term was to follow a CAR distribution, that is :

$\alpha_\mathrm{k}(\mathrm{A}_i) = \alpha_k(\mathrm{A}_i) + \varepsilon_k(\mathrm{A}_i)$, where $\alpha_k(\mathrm{A}_i) | \alpha_k(\mathrm{A}_j), i\sim j, \tau_k \sim N(\frac{1}{n_i}\sum_{i\sim j}\alpha_k(\mathrm{A}_j), \frac{1}{n_i\tau_k})$ indicating with $i\sim j$ the neighborhood relation between area A_i and A_j and $\varepsilon_k(\mathrm{A}_i) \sim N(0,1/\tau_{k\varepsilon})$.

The same model was done for the adjusted model as $\mathrm{logit}(\pi^*{}_{i'}) = \boldsymbol{\beta}^T X_{i'} + \alpha_1(\mathrm{A}_{i'})\mathrm{ITNownership}_{i'} + \alpha_2(\mathrm{A}_{i'})\mathrm{ITNuse}_{i'} + u_{i'} + v_{i'}$.
